# High rates of male courtship in a female-ornamented pipefish

**DOI:** 10.1098/rsos.231428

**Published:** 2023-10-25

**Authors:** Fleur van Eyndhoven, Elissa Z. Cameron, Sarah P. Flanagan

**Affiliations:** School of Biological Sciences, University of Canterbury, Private Bag 4800, Christchurch 8140, New Zealand

**Keywords:** Syngnathidae, sexual dimorphism, sexual conflict, sexual selection, courtship displays, *Stigmatopora nigra*

## Abstract

In species with sex-specific signalling traits that appear to be ornamental (i.e. are conspicuous and with no obvious natural selection benefit), the ornamented sex typically initiates courtship and is most active in courtship. Here, we report for the first time courtship displays in the extremely sexually dimorphic, female-ornamented wide-bodied pipefish (*Stigmatopora nigra*), revealing unexpected behaviours. Females use their sex-specific ornament during courtship displays, as expected, but rarely in female–female interactions. Surprisingly, males initiated 61% of reciprocated courtship bouts and chased females in 17% of the bouts. This chasing behaviour could be a form of male harassment or be indicative of female disinterest in ardent males, either of which was unexpected to be found in this female-ornamented species. Our results highlight the need to study the details of species' behaviours in considering the potential roles of sexual selection and sexual conflict in shaping sexual dimorphism.

## 1. Introduction

Sexual selection favours traits that increase the ability to gain access to fertilizations [1], such as pre-copulatory displays like ornaments or armaments and post-copulatory competitive traits [2]. A great deal of research has gone into understanding the mechanisms impacting the strength of sexual selection (recently reviewed in [3–5]), with key ideas focused around the importance of parental investment [6], operational sex ratios [7] and potential reproductive rates [8]. Nonetheless, the predominant views tend to centre on the so-called 'Darwin–Bateman paradigm', which posits that when sexual selection is stronger in males than females, (a) males have, in general, more variable reproductive success than females; (b) each additional mating provides larger fitness gains (i.e. more additional offspring per mating) for males than females; and (c) males are generally eager to mate and relatively indiscriminate, whereas female are more discriminating and less eager [1,9,10]. These basic principles are the foundation upon which current methods of estimating the strength of sexual selection are founded (reviewed in [11–14]).

The third prediction is the element of the paradigm that enables the evolution of traits by sexual selection, as the prediction that females are less discriminating is the method through which selection can act on male traits. Measuring eagerness to mate and mate preferences can be challenging, as these behaviours are influenced by factors that differ among environments and which can shift the trade-offs made by individuals of either sex, often in ways that are not straightforward [15]. Some trade-offs arise from biological constraints, such as previous investment into reproduction such as anisogamy [16] (but see [17,18]), the latency between matings due to reproductive requirements such as pregnancy [15], or costs associated with potential cuckoldry [19]. Other key factors can be more variable, such as the availability of mates in the population (i.e. the operational sex ratio) [20,21] or population density [e.g.^22^,^23^]), both of which shape the competitive environment for sexual selection. Furthermore, behaviours are inherently plastic traits, and plasticity in mating behaviours based on environmental conditions can impact mate availability over generations or even over the course of a season [3].

Although the Darwin–Bateman paradigm is articulated with males as eager and females as discriminating, examples exist wherein females experience stronger sexual selection, and these examples were in fact pivotal to helping Darwin and others articulate the predictions for sexual selection [3]. For example, several bird species (e.g. phalaropes, jacanas) display male brood care and females compete over access to mates, fish such as pipefish and some gobies display female competition over males, and several insects such as honeylocust beetles and dance flies show female competition over nuptial gifts (reviewed in [3]). Bateman's principles have been tested in some of these species and quantitative predictions that females have more variable reproductive success (prediction (a) above) and increased pay-off per mating (prediction (b) above) have generally been supported [24–28], with these metrics being used to identify if females in these species experience stronger sexual selection than males. This reversal of sexual selection strengths is recommended to be used as a way to define species as ‘sex-role reversed' [3,12,26], although this term has deservedly been criticized [3,29]. The extent to which the third prediction holds in these sex-role reversed species is perhaps less clear, especially as many studies pre-identify species as role reversed based on broad patterns of behaviour that sometimes reflect these mating behaviours but often instead refer to parental care [29]. Furthermore, we know that variation among individuals in mating behaviours is widespread [2,30] and that mutual mate choice can be beneficial for both sexes [31]. Therefore, investigating the extent to which males and females fit into these defined categories of roles is crucial.

Here, we investigate the third element of the Darwin–Bateman paradigm—eagerness in courtship—in a female-ornamented species, the wide-bodied pipefish (*Stigmatopora nigra*). This pipefish species has male parental care and sexual dimorphism in size, body shape and coloration, with females bearing a colourful ornament, which is hypothesized to be sexually selected [32] and used to attract males [33]. We investigate whether males and females differ in their ‘eagerness to mate' by describing courtship behaviours of males and females in experimental breeding populations. Specifically, we ask four questions: (i) What are the intra-sexual and inter-sexual behaviours, and do the sexes differ in which behaviours are most commonly used? (ii) Which sex initiates courtship most frequently? (iii) Do the sexes differ in the duration of their courtship displays? (iv) Does the size of a courting group impact courtship displays in both or either sex?

## 2. Methods

### 2.1. Study species

The wide-bodied pipefish is found throughout New Zealand and Australia [34,35] and is one of the most abundant species in Australian seagrass beds [36–39]. Females of this species have a dorsoventrally flattened belly with light and dark parallel stripes, an ornament which is correlated with fecundity ([32]; table 1 for images). Previous work identified the species as displaying female competition for mates based on the presence of this female ornament [33,40] and inferred sequential polyandry due to a larger number of mature eggs being present in female ovaries than numbers of developing embryos in male brood pouches [33]. Males and females were found in roughly equal numbers with a slight male bias in summer in an Australian seagrass bed [33], but the adult sex ratios in the New Zealand populations, which are substantially genetically diverged from Australian populations [41], are unknown.

**Table 1.** Ethogram of active and non-active courtship behaviours. Photography © Emily Beasley and Sarah Flanagan.

behaviour name	description	image	sex displaying the behaviour
wiggle	Side-to-side movement of the upper half of the body, displayed facing the recipient in a vertical position.	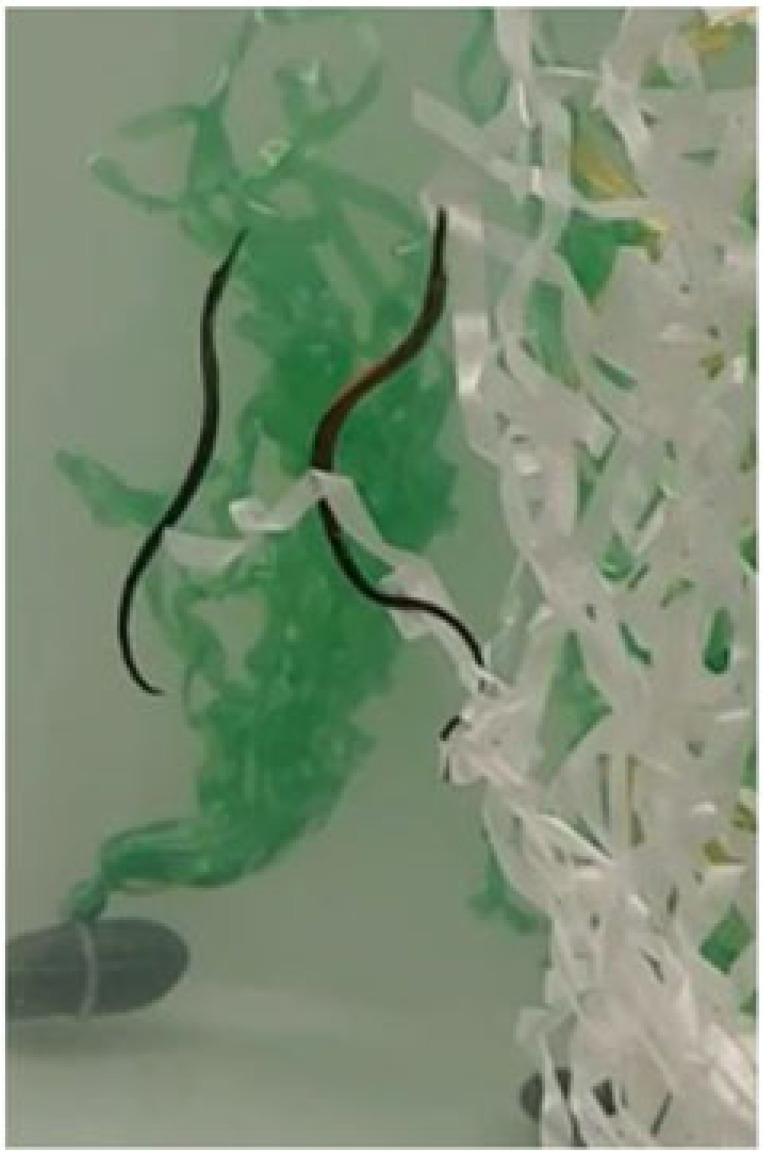 On the left: male (left) wiggles while female (right) poses.	males, females occasionally
pose	Female arches her back causing the belly to round forward, creating an S-shape. This ensured that the ornament was in full display towards the recipient.	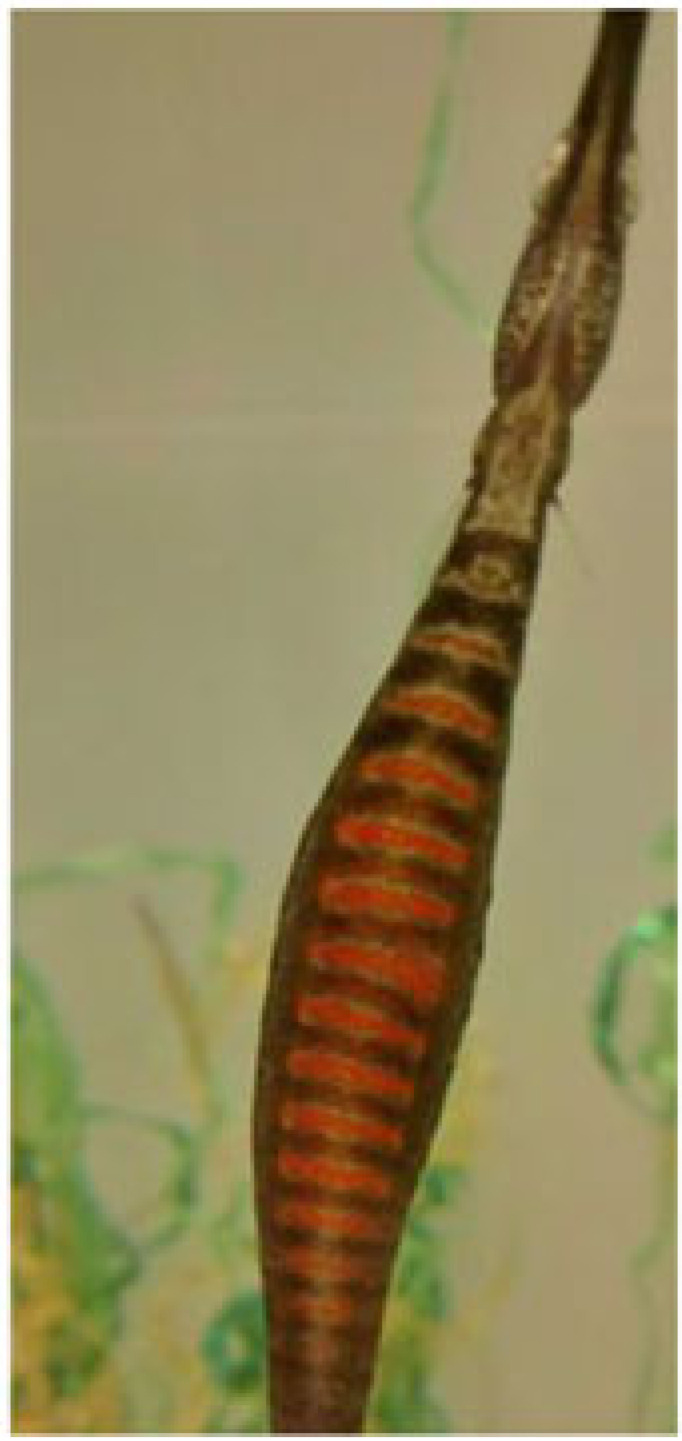	females
inactive	Pipefish do not display active courtship behaviours but remain close together in courting group.	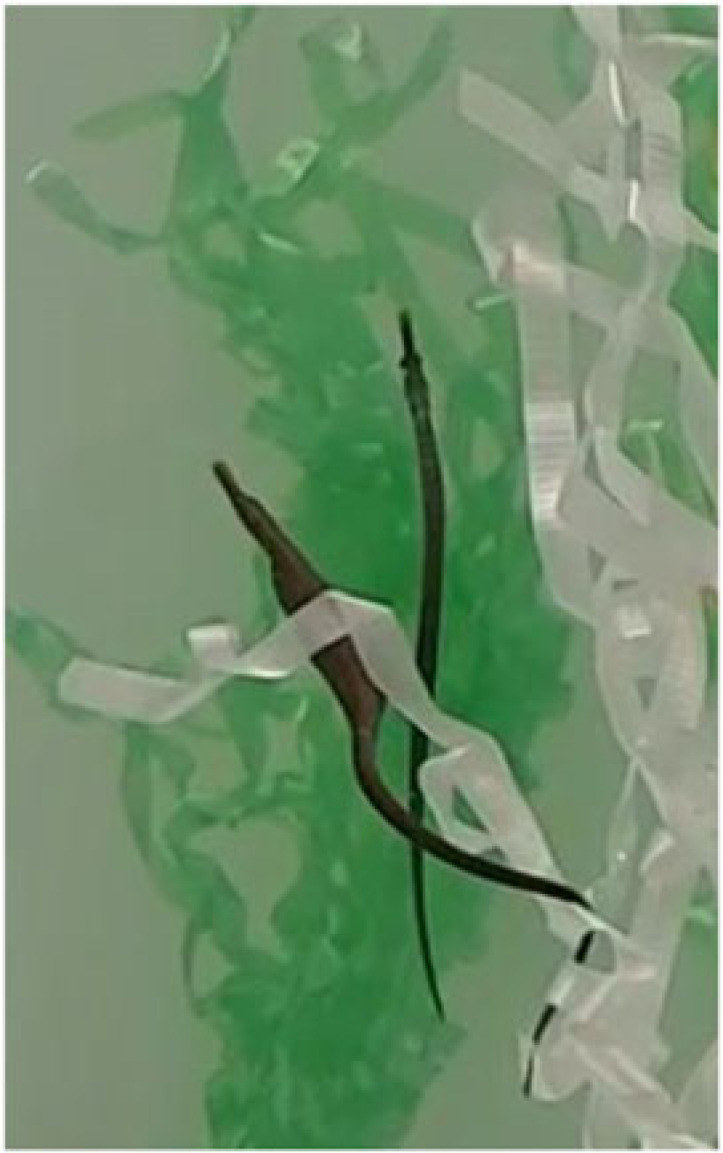	males, females
chasing	Males were observed swimming parallel to the female with speed.	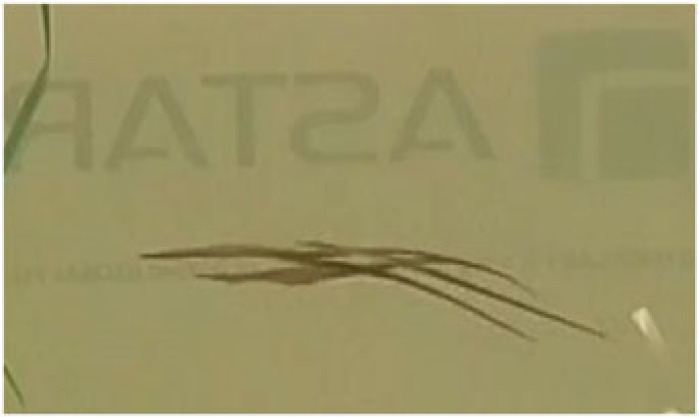	males
surrounding	Pipefish are surrounding the individual of the opposite sex (e.g. multiple males surrounding a single female).	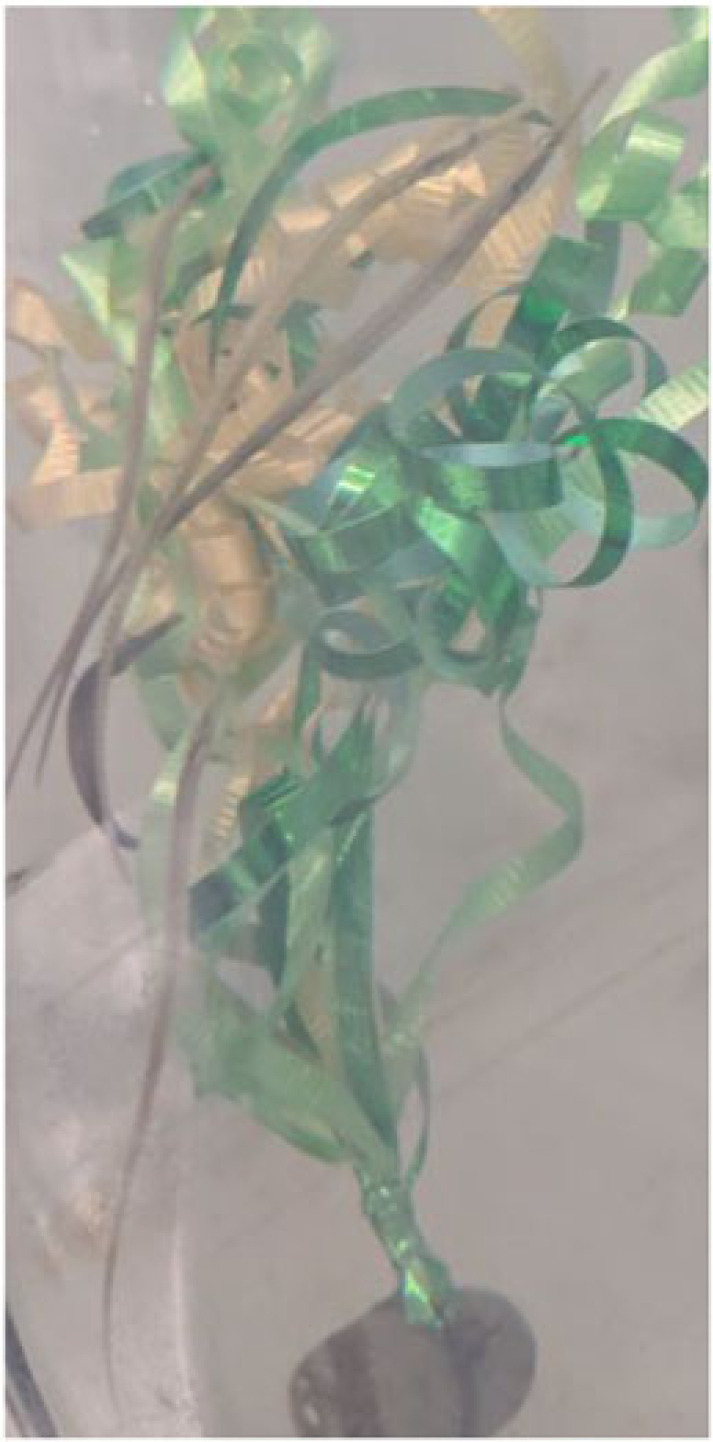	males

### 2.2. Pipefish experimental breeding populations

We collected pregnant male and ornamented female *S. nigra* (to ensure fish were sexually mature) in November 2020 at two sites in Tauranga harbour (North Island, New Zealand): 38 females and 60 males from Matua (latitude −37.66255, longitude 176.10827) and 62 females and 60 males from Plummers Point (latitude −37.66173, longitude 176.04525); individuals from both sites were mixed together for the study. Fish were caught using a 1.2 × 1.2 m push net with 1 mm mesh, housed overnight in flow-through tanks at the University of Waikato Marine Ecology lab, and transported to the University of Canterbury. Pipefish were then acclimatized to flow-through aquaria in 15 plastic 90 l tanks with white plastic taped to either side of the tank to act as a visual barrier between neighbouring transparent tanks (as in [42]). Pipefish were held at 18°C with a 12 h daylight cycle, comparable to conditions at the time and site of collection, with artificial seagrass as refugia, with a diet of day-old enriched *Artemia* spp. nauplii, provided twice daily, and supplemented by additional wild-caught zooplankton when available (approx. once per week). Tank water was filtered through biofilters, with 10–20% water changes performed daily. Males and females from each site were housed separately until all males had given birth (similar to [43,44]). We established 10 experimental breeding populations, each containing eight female and eight non-pregnant male pipefish, which experienced the same feeding and cleaning conditions as holding tanks. An equal sex ratio is consistent with wild populations [33] and densities are a little bit higher than capture rates in small patches of seagrass beds in natural populations [45] (though higher than densities on a large scale; e.g. [46]), and consistent with the numbers of fish captured in a single seine net pull when collecting the fish for this study. Prior to entering the experimental breeding populations, all pipefish were photographed and body size (total length) measured in ImageJ [47]. The average size of fish in each experimental breeding population was compared using a two-factor ANOVA (trial-by-sex). Experimental breeding populations were filmed daily for 7 days using a single Panasonic HC-V180 camcorder each from 8.00 to 10.00, when other syngnathids are known to have increased courtship [48–51], and 12.00 to 13.00 to capture fully diurnal behaviours.

### 2.3. Courtship behaviour analysis

Courtship bouts were scored using BORIS v. 7.10.5 [52] for exact counts and durations of observed behaviours (table 1). Courtship bouts were defined as groups of individuals displaying courtship behaviours and included individuals within two body lengths (snout-to-tail) of courting individuals. The initiating sex was identified by recording which sex performed the first active courtship behaviour (pose or wiggle; following [48]). The arrival of new individuals into the group (within two body lengths) and the departure of individuals were tracked and average group size over the duration of each bout was calculated. Courtship bouts were considered finished when pipefish in the courting group had displayed non-active behaviours for greater than 60 s or if courting individuals had moved more than two body lengths (snout-to-tail) away from each other. For each behaviour observed, the sex of the displaying individual was recorded. For active courtship behaviours (poses and wiggles), the sex of the fish directly in front of the displaying individual was recorded as the receiving sex. Behaviours were scored by sex as individuals could not be individually identified in the videos. Owing to this constraint, the bouts include repeated measures of individuals within trials and are unable to reflect variation among individuals. To account for the non-independence of events, all statistical analyses include trial number as a random effect (see below).

All statistical analyses were conducted in R v. 4.0.5 [53]. The proportion of reciprocated courtship bouts initiated by males was compared with a null expectation of 0.5 using a proportion test. To determine whether females displayed their ornament more frequently during inter-sexual or intra-sexual interactions, a proportion test was used to compare the proportion of female displays performed towards males with 0.5. The frequency of unreciprocated courtship events was compared between the sexes using a *χ*^2^-test.

We performed model selection of linear mixed models of the log-transformed durations of active courtship behaviours to identify which factors were most important in predicting the duration of active courtship behaviours. The fixed effects tested were sex of the individual displaying, the size of the group when the display was performed, the time of day (morning or noon), the day in the trial when the behaviour was recorded (days 1–7) and the total time of courtship in the bout. Bout numbers nested within trial were included as random effects. Only reciprocated courtship bouts (i.e. both sexes displayed active courtship) were included. Models with corrected Akaike information criterion (AICc) scores that differed from the best-scoring model by 5 or fewer were further analysed and their estimated means interpreted. We used MuMIn [54], lme4 [55], lmerTest [56] and emmeans v. 1.7.2 [57] to conduct this analysis and perform *post hoc* tests.

We predicted the probability that a courtship bout included chasing using a generalized linear mixed-effects model with a binomial logit-link function, implemented with lme4 [55]. Group size, bout duration and their interaction were included as predictor variables, with the time of day and trial number included as random effects. We fit an equivalent model to predict the probability of a bout ending in chasing (i.e. the final behaviour was chasing), but instead used a cloglog link function using VGAM [58,59] and back-transformed fixed-effects estimates. We tested for a correlation between the proportion of bouts each trial that contained chasing and the overall difference in female body lengths (i.e. amount of female variation) using a Spearman rank correlation.

## 3. Results

Experimental breeding populations did not differ in the average size of male or female pipefish (*F*_9,9_ = 0.311, *p* = 0.97), although females were larger than males (*F*_9,1_ = 33.649, *p* < 0.001; average male: 93.40 ± 0.94, average female: 101.84 ± 1.06). Twenty-six males became pregnant in the experimental breeding populations (32.5% of all males across trials, and greater than or equal to 1 pregnancy per trial), although copulation was never recorded. Most trials experienced some mortality (average 1.5 males, 1.7 females per trial), but most deaths occurred after the filming was completed (i.e. after the first week of the experimental breeding population). We recorded, in total, 22.58 h of courtship bouts, which contained a total of 9.01 h of active behaviours (other than ‘inactive' or ‘out of sight'), including poses, wiggles, chasing and surrounding (table 1 for descriptions). Courtship bouts lasted from 12.22 s to 50.03 min (mean = 3.47 min). Poses and wiggles were the most common active behaviours (83% of active behaviours) followed by chasing and surrounding (12% of active behaviours). Poses and wiggles were considered active courtship behaviours for the remaining analyses.

**Figure 1.**
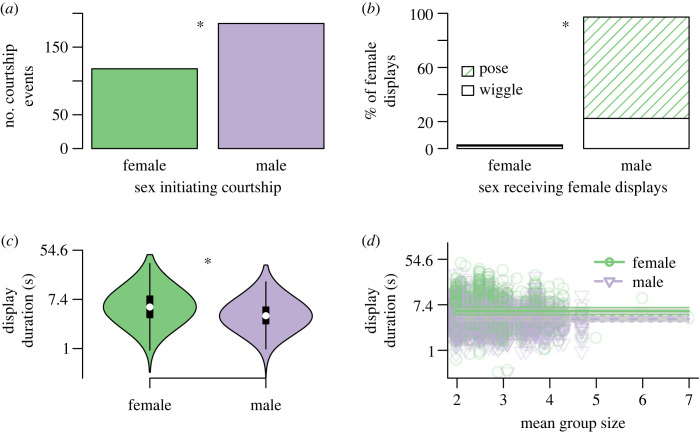
Courtship behaviours differ between the sexes. (*a*) Males initiated most courtship displays, all of which were directed towards females. (*b*) Female displays were primarily directed at males, and females primarily used poses instead of wiggles. (*c*) Females had longer durations of displays than males, with a higher median (white dot) duration time. Box plots within the violins show the interquartile range with lines extending to the 1.5× the interquartile range in both directions. (*d*) Displays are slightly longer in large groups for both males and females. All groups consist of a single female. The lines show the linear relationship (with the 20% and 80% quantiles around the predicted durations shown) between duration and group size for males and females separately. Note the log-scaled *y*-axis in *c* and *d*. Asterisks indicate significant differences between sexes.

Of 391 courtship bouts scored, 77.5% were reciprocated. Males initiated 61% of these reciprocated bouts, significantly more than females (proportion test: $\chi_{1}^{2}=14.38$, *p*-value < 0.001; figure 1*a*). Females displayed their ornament significantly more frequently towards males than females (proportion test: $\chi_{1}^{2}=1306.5$, *p*-value < 0.001), and 97% of female displays were directed towards males (figure 1*b*). Only 3% of female displays were directed at females, of which 68% were wiggles and 32% were poses. Groups of displaying fish ranged from 2 to 7 individuals, with an average size of 2.54 individuals. All groups contained at least one female, and all groups comprising more than two individuals included only one female (i.e. non-dyad groups consisted of one female and two or more males).

Five models of active courtship durations had a ΔAICc ≤ 5, all of which included the sex of the displaying individual. Three of the top models included group size and two included time of day (table 2). None of the top-ranked models included day of filming or total bout length. The best-scoring model included only sex and the random effects (bout number nested within trial; ‘model 1', AICc = 5579.83, d.f. = 5; electronic supplementary material, table S1), with the second-best model including sex and group size and the random effects (‘model 2', ΔAICc = 1.12, d.f. = 6; electronic supplementary material, table S1). In all top models, female displays were longer than male displays (table 2 and figure 1*c*). Larger groups had lower durations per display behaviour (table 2 and figure 1*d*), although the effects are an order of magnitude smaller than sex effects (table 2).

**Table 2.** The estimated marginal means (EMM) for male and female active courtship behaviours from the top five models, estimated separately for morning or noon filming times for models including time of day as a factor.

model	log likelihood	AICc	Δ AICc	female EMM ± s.e.	female d.f.	male EMM ± s.e.	male d.f.	time of day	group size est.
subject + (1|trial/bout)	−2784.9	5579.827	0.000	1.658 ± 0.042	8.831	1.311 ± 0.041	8.207		n.a.
subject + group size + (1|trial/bout)	−2784.46	5580.95	1.124	1.657 ± 0.038	8.820	1.310 ± 0.038	8.063		−0.063 ± 0.025
subject × group size + (1|trial/bout)	−2784.97	5583.975	4.148	1.658 ± 0.038	8.814	1.310 ± 0.038	8.065		−0.097 ± 0.030
subject + group size + time of day + (1|trial/bout)	−2786.26	5584.536	4.709	1.675 ± 0.040	10.254	1.326 ± 0.039	9.316	morning	−0.065 ± 0.025
				1.620 ± 0.045	15.219	1.271 ± 0.045	14.570	noon	
subject + time of day + (1|trial/bout)	−2785.59	5585.22	5.393	1.674 ± 0.044	10.006	1.326 ± 0.043	9.244	morning	n.a.
				1.623 ± 0.048	14.219	1.276 ± 0.048	13.695	noon	

**Figure 2.**
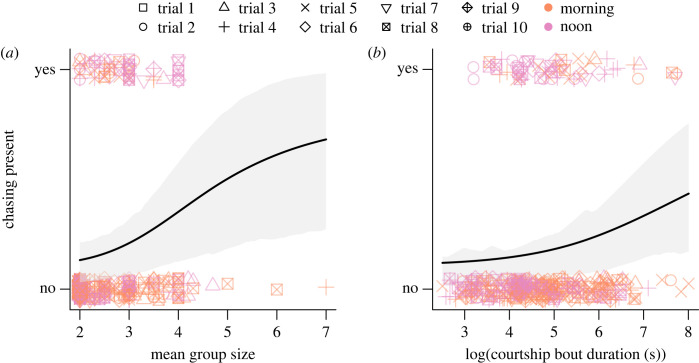
Chasing occurred more frequently in longer bouts with large groups. Shown are the observed presence or absence of chasing with some jitter added across the mean group size within each bout (*a*) and the log of the duration of the bout (*b*). Point shapes represent trials and colours show the time of day the bout occurred, which were the random effects in the logit generalized linear mixed model. Grey shading represents the 20% and 80% quantiles around the predicted values. All groups consist of a single female.

Sixty-six of the 391 courtship bouts included chasing behaviours (16.88%). Of the bouts with chasing, 56.06% had chasing as the final behaviour. Without the inclusion of the group size or bout duration, bouts were unlikely to include chasing, with an estimated less than 0.001% [95% CI: 0–0.0003%] chance of chasing occurring (*β* = −14.04 ± 2.96, *z* = −4.74, *p* < 0.001). The inclusion of group size increased the probability of chasing to 98.18% [95% CI: 86.89%–99.77%] (*β* = 3.99 ± 1.07, *z* = 3.73, *p* < 0.001), and the inclusion of duration alone increased the probability of chasing to 89.86% [95% CI: 73.56%–96.57%] (*β* = 2.18 ± 0.59, *z* = 3.69, *p* < 0.001). Including both group size and bout duration increased the probability of chasing occurring by 33.65% [95% CI: 24.67%–43.99%] (*β* = −0.68 ± 0.22, *z* = −3.04, *p* = 0.002). Larger groups and longer lasting bouts therefore increased the probability that a bout would include chasing (figure 2). However, the probability of a bout ending with chasing as the final behaviour could not be predicted by group size (*β* = 3.26 ± 2.05, *z* = 1.59, *p* = 0.112), duration (*β* = 1.86 ± 1.22, *z* = 1.53, *p* = 0.127), or their interaction (*β* = −0.71 ± 0.45, *z* = −1.59, *p* = 0.112). Chasing tended to occur in trials with larger differences in length between the longest and shortest females, but the relationship was not statistically significant (*ρ* = 0.558, *p*-value = 0.094). Owing to study design, the size of individuals performing behaviours was unable to be measured.

## 4. Discussion

We describe for the first time the behaviours associated with courtship displays in the wide-bodied pipefish (*Stigmatopora nigra*). Two key courtship behaviours were observed—poses and wiggles—which were displayed with different frequencies in males and females. Males only wiggled, and always wiggled towards females, whereas females displayed both poses and wiggles towards males, and very rarely towards other females. Our results demonstrated that females use their ornament in active courtship displays towards males, but not in competition with other females, providing behavioural data to support a previously hypothesized use of this sexually dimorphic trait [33]. Females were generally more active in courtship than males, with longer display durations than males. However, males initiated courtship more frequently than females and both sexes displayed for shorter durations in larger groups of courting individuals. Furthermore, we documented an inter-sexual chasing behaviour performed by males, which was associated with larger courting groups and longer courtship bout durations. These observations portray nuanced patterns of behaviour consistent with both sexes displaying active courtship and probably expressing mutual mate choice.

The wide-bodied pipefish did not follow the dichotomized sex roles predicted by the Darwin–Bateman paradigm predictions. Females were ornamented, displayed multiple courtship behaviours (both wiggles and poses), and displayed for longer durations within courtship bouts than males, but they initiated courtship bouts less frequently than males. Given that the ornament reflects fecundity in natural populations of *S. nigra* [32], males might use the ornament as an honest signal of offspring quality and/or brood size, as seen in other pipefish [60–63], and males probably initiated courtship with females with the largest ornament—but their high frequency of initiation suggests that males are both eager and discriminating. We observed chasing behaviours in 17% of courtship bouts, which can be interpreted in two ways: males use chasing behaviours to assess female quality, or chasing occurs when a female does not want to pursue courtship further but males want to continue courting the female. Regardless of the motivation for chasing, combined with the male initiation, both males and females are clearly eager in courtship.

The courtship dynamics observed here could be explained by several factors that are not mutually exclusive. One factor is that male pipefish are removed from the pool of mating individuals after becoming pregnant, which could impact our results in two ways: first, non-pregnant males might be very eager to re-mate to maximize their lifetime reproductive success, similar to how long ‘dry time' impacts sexual selection in theoretical models [15] and second, the operational sex ratio will shift in the experimental breeding populations as males become pregnant. In this second scenario, the operational sex ratio would shift to be female-biased, which in other pipefish can result in stronger sexual selection on females [22], stronger male mate preferences [64], fewer overall associations between males and females [65], and increases in female–female interactions [65,66]. We did not observe any aggressive interactions, including disruption of courting pairs as seen in other pipefish [50,51,67], and our best-fitting models of courtship durations showed no difference between day 1 and day 7, so we do not have substantial evidence that small shifts in operational sex ratio due to males becoming pregnant had a major impact on the behaviours observed here.

Although the mesocosms were initialized with equal adult sex ratios, with presumably sexually mature fish, multiple males frequently courted a single female in groups, which raises questions about why these groups form. One possibility is that males are choosy based on some threshold for attractive female ornaments (or female sizes), so only a subset of females were sufficiently attractive—probably the large, ornamented and active females [42,48,68–73]—resulting in a male-biased realized operational sex ratio in the breeding tanks. In other pipefish, groups of multiple females can interrupt a courting pair [50,67], so it is possible that groups are a way for males to compete with each other over the most attractive females. This could be similar to a lek, although in most species the ornamented sex is the one that displays in groups [74] (including in a pipefish, [75]), and none of our groups included more than one female. Alternatively, males could perform mate copying, a phenomenon observed in *Syngnathus typhle* [76]. A rich area for further study would be to investigate the plasticity of the nuanced roles we have discovered, and for example see whether the composition of the groups change in response to shifting operational sex roles over the course of a breeding season [63,66] or the availability of resources in the environment (e.g. [77]).

The active roles of both sexes in courtship hint at possible evolutionary conflict over mating rate in this species (i.e. inter-locus sexual conflict). Our finding that females display longer than males could imply that a single courtship bout might be more energetically costly for females. Additionally, the chasing behaviour could also negatively impact female fitness and behaviour, similar to how male harassment of females decreases female fitness (e.g. [78,79]) and can alter female movement and dispersal [80] in species with conventional sex roles. If these costs do not trade off with other components of fitness, the two sexes might have different optimal mating rates in this species, which could result in sexual conflict. Further work is required to identify the costs and benefits to both sexes of this chasing behaviour, and how consistently these behaviours are displayed in different contexts, for example, female-biased operational sex ratios or lower densities of fish.

In other female-ornamented species, females initiate courtship [51,69], but we found evidence for more nuanced sex roles, with females being ornamented and males ardent in courting. These nuanced sex roles are consistent with complex patterns of behaviours described for other sex-role reversed species [81,82]. We also document male chasing behaviours for the first time in a pipefish, which could indicate that sexual conflict over mating rates or other courtship behaviours is occurring. Further work is needed to establish how these behaviours respond to ecological and environmental factors, and their associated direct and indirect fitness costs. While ornamentation does not appear to trade off with fecundity in this species [32], it could have trade-offs in energetic costs if those females are spending more energy courting or avoiding courtship by males. We find more nuanced patterns of mating behaviours than predicted by the Darwin–Bateman paradigm for a female-ornamented species.

## Data Availability

The raw data are archived on Zenodo: https://zenodo.org/record/7735430 [83]. The code and processed data used for the analyses presented in this manuscript and creation of tables and figures is available at github: https://github.com/flanagan-lab/Snigra_behaviour/releases/tag/published-v.1.0, which is archived on Zenodo: https://doi.org/10.5281/zenodo.10005680 [84]. Table S1 is provided in the electronic supplementary material [85].
